# Reviews and Book Notices

**Published:** 1878-11

**Authors:** 


### Reviews and Book Notices.

Handbook of Ophthalmology. By Prof. C. Schweigger, of the University of Berlin. Translated from the third German edition by Porter Farley, M. D., Rochester, N. Y. Philadelphia: J. B. Lippincott & Co. Chicago: Jansen, McClurg & Co.

We cannot imagine what has induced the translator to omit the author's preface. It is certainly contrary to all usage, and especially in this case we regard this omission a strange mistake — the only mistake, by the way, Dr. Farley can be accused of—because Schweigger's book differs from the usual plan and style of text-books so essentially, that the explanatory preface of the author is indispensable to an understanding of his intentions and his idea of a " handbook."

Schweigger has no great faith in mere book learning; he does not believe that a text-book can be substituted for didactic lectures and clinical demonstrations; he regards a handbook merely as the skeleton to which clinical teaching can and must give flesh and life. He, therefore, endeavored to make his book as concise as possible, and on this account omitted an introductory chapter on anatomy and physiology of the eye. With reference to this omission he says in the preface to the first German edition: " He who wishes to engage in the study of ophthalmology must possess the requisite knowledge of anatomy and physiology and optics, or he must be able occasionally to consult the text-books of these disciplines. Only where the discussion of certain questions required it, the field of the above named branches was entered upon." We fully indorse every word. These chapters on the anatomy and physiology usually found in text-books, are too generally incomplete to be of much value, and only fill a number of pages which may be used to a better purpose. The omission of these chapters inaugurates a reform in the right direction; it places the ophthalmological text-books on the same standard with works on surgery or practical medicine, which do not contain the anatomy and physiology, but only discuss the diseases of the several parts of the human body.

In view of the fact that within seven years Schweigger's handbook has attained its fourth edition, it would seem almost preposterous to question if the English edition will meet with a similar success. It has many excellent features, not the least of which is the absence of all unnecessary phraseology. The style is very compact; the descriptions are condensed into the fewest possible words. But the desire for utmost conciseness is carried to an extreme; the author is often so brief, his writing so aphoristic that the reader must already have a great deal of knowledge in order to catch the meaning of the text. This is not the right style of a book for students, and we therefore fear it will not become a popular text-book in this country.

Besides it seems to us as if the work does not show that keen appreciation for the wants of students and practitioners, which is always a prominent feature of English text-books. A glance at the general composition will show the difference between these and the German work. Schweigger has divided his book into three parts of almost uniform length. The first part treats of the anomalies of refraction and accommodation, and of the ocular muscles; the second part contains the diseases of the orbit, lachrymal apparatus, eyelids, conjunctiva, and all affections of the eyeball, except those of the fundus; and in the third part the diseases of the fundus (choroid, retina, optic nerve, glaucoma and amblyopia) are discussed. The first part covers 188 pages; the second part, 210 pages, and the third part, 148 pages. Soelberg Wells allows to the same subjects 110, 383, and 112 pages respectively; Brundewell Carter, 85, 245 and 60 pages. These and other authors justly devote by far the larger space of their books to the consideration of those diseases which are common in the daily practice of a physician and on which he needs the most information. Schweigger disregards these practical wants, and seems determined to make his three parts as uniform as possible, just as if he had written a drama under the old rule that the three acts should be about equal in length. In order to accomplish this aim he launches in the first part upon theoretical speculations which are just as learned as they are useless. For instance, he devotes ten pages to a discussion on the size of the ophthalmoscopic image. The mathematical formulæ in which these calculations abound, will make a student's hair stand on end. But *cui bono?* What practical results can be drawn from these calculations? None, whatever! The very foundation of the formulæ is hypothetical, and consequently the result must be liable to gross errors. The calculations are based upon the assumption of an uniform size of the optic disc, and upon the optical relations of the Listing-Donders' schematic eye. But the optic disc is not of an invariable size; it shows individual variations. And the schematic eye does not accurately represent the dioptric apparatus of the innumerable individual variations of the human eye. But granted the ophthalmoscopic image to be enlarged from fifteen to twenty times; does it so appear to the observer? If we let a dozen students make an accurate drawing of the same optic disc as it appears to each of them, we shall observe the strange fact that no two students will represent the same size of the optic disc. And we only need to compare the ophthalmoscopic pictures of a Stellwag, a Jaeger, a Liebreich and others in order to be fully convinced of the fact that the enlargement in which the fundus appears to each observer, depends as much on his individual faculty of projection as on the magnifying power of the dioptric media. Jaeger, for instance, is known for the scrupulous accuracy and enduring pains with which he delineated the pictures of his ophthalmoscopic atlas; yet all his figures appear as if magnified seven times only! And to us at least the optic disc always appears larger than Jaeger's figures.

If we finally mention that Schweigger himself admits that no practical conclusions can be drawn from these calculations, we hope the reader will agree with us that such useless theoretical speculations do not enhance the practical usefulness of a book.

Still this is a very slight blemish as compared with the sometimes very unsatisfactory way with which practically important subjects are disposed of in the second part of the book. To repeatedly advocate the use of lead lotions without a word of caution as to the damage which may possibly be done to the cornea by staining; the dismissal of so important a disease as iritis on a few pages; and to say not one single word about the division of the cataract operation into the preliminary irridectomy and the extraction (which plan has been adopted by many surgeons as the safer procedure), these are only a few instances of the deficiencies which we believe seriously effect the practical value of the book.

The translation and publishing work is done with great skill and praiseworthy care. We have discovered only one error worth mentioning (which by the way is not found in the original): on the last line of page 41 it should read 12 inches for 4.

F. C. H.

TRANSACTIONS OF THE AMERICAN GYNÆCOLOGICAL SOCIETY. Volume II, for the year 1877. Boston: Houghton, Osgood & Co.; Chicago: Jansen, McClurg & Co.

This most elegant and attractive work has but lately reached us, after many months of expectation; so long, in fact, had the reviewer waited for its appearance, that he had determined to shut his eyes to its external beauty, enter the book boldly, and slay a few of its contributors at all hazards. Now that it is here, it disarms adverse criticisms by its real excellence.

To begin with, there is the address of the retiring President, Fordyce Barker, in which he holds up the finger of warning to the over-bold modern uterine surgeon, and deals out to the members much advice in his usual dignified manner. Space not permitting, we can only pick out and mention some of the most prominent among the many original papers which follow.

First among these comes a paper on "The Functions of the Anal Sphincters," by James R. Chadwick, the secretary of the society, in which one only needs to read of the numerous experiments of the author to recognize the conscientiousness of his search after facts.

By special request from the society, John C. Dalton, of New York, presented a "Report on the Corpus Luteum," with twelve chromo-lithographic plates of extreme beauty, which gives us a pretty good idea of our present knowledge of these bodies, and makes permanent the idea of them that could only be outlined in the descriptive part of the report.

Following this is an essay on the "Pathology and Treatment of Puerperal Eclampsia," by Professor Otto Spiegelberg, of Breslau, Prussia, and another on "Dilatation of the Cervix Uteri for the Arrest of Uterine Hemorrhage," which is simply the reiteration of a fact long known and established, and cannot be styled original.

Of no small value is the warning of William T. Lusk, of New York, "On the Necessity of Caution in the Use of Chloroform During Labor." There can be no doubt that many cases are placed on record as death from syncope, or hæmorrhage, or heart disease, or pulmonary thrombus, etc., that, if clearly sifted, could be traced directly to the employment of chloroform. Dr. Lusk, after a candid recital of several unfortunate cases that came under his direct observation, sums up his experience as follows:

1. Deep anæsthesia carried to the point of complete abolition of consciousness, in some cases weakens uterine action, and sometimes suspends it altogether.

2. Chloroform, even when given in the usual obstetrical fashion, namely, in small doses during the pains only, and after the commencement of the second stage, may, in exceptional cases, so far weaken uterine action as to create the necessity for resorting to ergot or forceps.

3. Patients in labor do not enjoy absolute immunity from the pernicious effects of chloroform.

4. Chloroform should not be given in the third stage of labor. The relative safety of chloroform in parturition ceases with the birth of the child.

5. The remote influence of large doses of chloroform during labor, upon the puerperal state, is a subject that calls for further investigation and inquiry.

Following this comes what was evidently the prominent discussion of the meeting, suggested by a paper from Ely Faw-de-

Warker, of Syracuse, "The Present Status of the Intra-Uterine Stem in the Treatment of Flexion of the Uterus," in which the doctor makes an able and carefully-worded plea for what is evidently with him a favorite mode of treatment for uterine displacements. His views, as expressed in that paper, gave rise to a very warm discussion, in which almost every member joined, it being quite evident that the author had touched a very sensitive spot in the recollection of most of those present.

Dr. Battey, of Rome, Ga., next propounds the query: "Is there a Proper Field for Battey's Operation?" in which he so ably and logically sustained the affirmative, that he received quite an ovation of praise from all who joined in the discussion following the reading of his paper.

Probably the effort entailing the most time, trouble and careful consideration was that of Dr. Paul F. Mundé, of New York, on "The Value of Electrolysis in the Treatment of Ovarian Tumors." It occupies eighty-nine pages of the book, and contains much valuable information, the result of many months of continuous labor on the part of the author. We notice in this paper a discussion on a doubtful case of ovariotomy between two Chicago surgeons. This part of the book ends with a treatise on the "Hystero-Neuroses," "with special reference to the menstrual hystero-neurosis of the stomach," by George J. Engelman, of St. Louis, most carefully and elegantly written.

The second portion of the "Transactions" is devoted to the publication of papers presented to the Council by the candidates elected to fellowship. These seem to be all relating to subjects of practical importance, and the mention of their titles will suffice to give the reader a very fair idea of their nature; 1st, "Cases Illustrating Important Points Connected with Ovariotomy," by Gilman Kimball, of Lowell, Mass. 2d, "The Radical Treatment of Dysmenorrhea and Sterility by Rapid Dilatation of the Canal of the Neck of the Uterus," by Ellwood Wilson, of Philadelphia. 3d, "Dr. Uredale West's Views of Rotation," by John P. Reynolds, of Boston. 4th, "Vascular Tumors of the Female Urethra, with the Description of a Speculum Devised to Facilitate their Removal," by A. Reeves Jackson, of Chicago. 5th, "The Simple Varieties of Perineal Lacerations; Their Prevalence, Consequences, and the Importance of Radical Treatment," by Thaddeus Reamy, of Cincinnati, in which the author rather startles us by the recital of the terrible consequences following any ununited rupture of the perineum, no matter how slight, and rather disturbs our conscience when we reflect on our guilt in not always doing as the doctor does, always restoring the perineum completely, even taking in a little more, when practicable, as he sometimes does. 6th, "Lying-in Institutions; Especially Those of New York," by Henry J. Garrigues, of Brooklyn; and 7th, "The Menstrual Cycle," by John Goodman, Louisville.

The paper on lying-in institutions is particularly interesting reading, and might prove of particular interest to the medical board of our County Hospital, who are at present countenancing the fitting up of a garret in a large general hospital for lying-in purposes.

The last pages of these "Transactions" are occupied by an index of gynæcological and obstetric literature of all countries, alone worth almost the price of the book. If any society of this kind has yet published a volume of transactions equal to this we have certainly never seen it.

THE PHYSICIAN'S VISITING LIST FOR 1879. Twenty-eighth year of its publication. Philadelphia: Lindsay & Blakiston.

As the first memento of the approaching new year, this little book has come to us. It is such a dear and true friend of every physician that our readers will undoubtedly be pleased to learn of its arrival. It looks as well as ever in a new coat and with all the old good qualities which have made it the pet of the profession.

ANATOMY, DESCRIPTIVE AND SURGICAL. By Henry Gray, F. R. S., etc. A new American edition, from the eighth n d enlarged English edition. To which is added Landmarks, Medical and Surgical, by Luther Holden, F. R. C. S. Philadelphia: Lindsay & Blakiston; Chicago: Jansen, McClurg & Co.

It would be superfluous to say more than to announce the appearance of a new edition of this excellent work, which for twenty years has been the most popular text-book of anatomy in the English language. The editors have carefully retained all the good features of the old editions and have made all the changes in the section of microscopical anatomy necessary to keep the standard of the book abreast with the advancement of microscopy. The addition of Holden's Landmarks will only enhance the practical value of the book.

How to be Plump: Or Talks on Physiological Feeding. By T. C. Duncan, M. D., etc., etc. Chicago: Duncan Brothers, 1878. 12 mo. pp. 60.

This little volume is a remarkable production. It goes forth as a tract to the people and the doctors, ostensibly to instruct the lean how to be plump.

Plain directions are given for the accomplishment of this purpose, in a most inexpensive manner. Thin down your diet to slops; eat soup freely, and drink regularly of water between meals—this is the way. Could anything be simpler?

But the book is clearly written for a double purpose. The object referred to above, is the one that will present itself to the understanding of the general reader; the other is more recondite, and will hardly be fully appreciated by any but members of the profession. This purpose—the one that will send this literary marvel down to history—is one of humor, of burlesque; and the victims the author's darts are aimed at, are the fathers of our physiology.

Much of the matter is quoted from standard works on physiology, and does not call for comment. It is only when we come to the original part of the work that we see the acuteness of the author's satire. From the latter we make a few quotations.

Regarding acid condiments, he says: "Acids of all kinds quicken the circulation, unduly excite the system, while they at the same time tend to break down the cells."

Referring to the relative composition of the blood in the different temperaments, we are told that "Lack of water produces a preponderance of red corpuscles in the sanguine."

Again, "Water is formed in small quantities in the body!!!"

"Many a case of spinal irritation and nervous exhaustion, are due (sic) to a lack of qantity and quality of the blood current, of which 80 per cent. is water."

A glass of water taken after a meal, forces "the food farther down the alimentary tract"—the digestive apparatus seeming to be regarded as an inelastic tube, and ingestion like the stuffing of sausages.

"Fat is found in nearly all parts of the body; it aids digestion and assimilation, quickens the circulation, and hastens cell activity."

"The importance of fat are (sic) physical, mental, and moral."

"In the lean, the functions are performed with difficulty; the digestion is feebly performed; friction is manifest everywhere, and there is often explosions (sic) of the nervous system, i.e., spasm, neuralgia, or bursts of passion."

"In two weeks it"—i.e., the baby—"ate the whole time." What an appetite!

"Starchy food taken in excess, taxes the liver till it becomes enormous in size from the deposit of fat therein. If persevered in, respiration is impeded, and there is great danger of suffocation." Here is a hint to the true solution of the Chinese question—feed John upon starch to suffocation.

"Too much water should be avoided, as it sends the blood too rapidly into the capillaries, and some of the serum transudes in the form of perspiration."

Before retiring for the night, a half glass of water should be taken, as it "insures quiet and refreshing sleep."

Truly the gift of this author is a rare one.

